# Use of suction catheter as an aid to intubation in emergency situation of intraoral bleeding

**DOI:** 10.4103/0019-5049.65358

**Published:** 2010

**Authors:** Mohammad Aseem, Urmila Palaria, Umesh Kumar Bhadani

**Affiliations:** Department of Anaesthesiology, Uttarakhand Forest Hospital Trust Medical College, Haldwani, Nainital, India

Sir,

Intraoral pathology poses a great challenge to the anaesthesiologists in terms of oral intubation. Endotracheal intubation is difficult because of restricted space for larygyscopy and non-visualisation of glottis. The problem gets compounded if the oral mass is vascular. We present a case where use of suction catheter helped in achieving oral endotracheal intubation in emergent situation.

**Figure 1:**
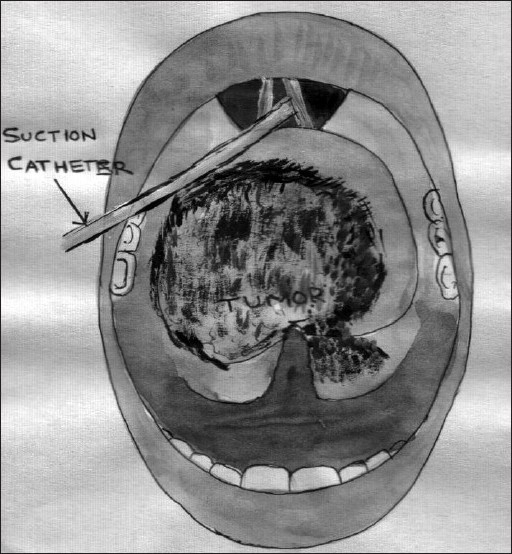
Diagrammatic representation of suction catheter in glottis and bleeding intraoral mass

A 37-year-old, ASA physical status II male, weighing 80 kg was posted for examination and biopsy of an intraoral mass under general anaesthesia. The patient was placed on operation table with monitoring for SpO_2_, non invasive blood pressure and ECG. He was a patient of difficult airway with intraoral mass, obesity and Mallampati score of III. Our institution does not have the facility of fibre optic intubation. Difficult intubation cart was ready with Laryngeal Mask Airway, light wand and other adjuncts of airway management. Endotracheal intubation by laryngoscopy was planned as a usual procedure. After mask ventilation with 100% oxygen and induction with thiopentone and suxamethonium, direct laryngoscopy was performed. Mouth opening was adequate; so, as practiced for routine procedure, thiopentone and suxamethonium were used for intubation. The glottis was visible but suddenly untoward happened. With the manoeuvring of laryngoscope, the intraoral mass started to bleed profusely. There was imminent risk of aspiration of blood by the patient. Suction was ready and suctioning was started. During suctioning, the glottis became visible. In this kind of emergent situation, suction catheter was deliberately inserted into the glottis and left *in situ* [Figure 1]. Oxygen was connected to this suction catheter. Another suction catheter was used for further suctioning. Distal end of the first suction catheter was cut and a cuffed oral endotracheal tube no. 8.0 mm was “railroaded” over it. Cuff of endotracheal tube was inflated and endotracheal tube was secured by tape. Oxygenation through first catheter was continued till the situation was under control. Suction catheter was taken out and the correct placement of the endotracheal tube was confirmed. From laryngoscopy to intubation it took about 4 minutes. Oxygen saturation and other hemodynamic parameters were within normal limits throughout the emergent situation. The patient was under anaesthesia during intubation attempt. Surgery was completed uneventfully.

Endotracheal intubation in a difficult airway is always a challenge to anaesthesiologist. The problem gets compounded if the difficult airway is due to bleeding mass in oral cavity.[1] Laryngeal mask airway is a useful adjunct of airway management in situation of intraoral mass.[2] Light wand and fibre optic intubation are an alternative but it needs prior expertise and some time may be a difficult alternative in emergent and active bleeding situation. In this case, even if adjuncts of airway were present there was no time to use them. The situation needed immediate action. Presence of mind and use of common and easily available resources may be life saving in certain situations as it was seen in this case report.
